# T225. OPERATIONAL DEFINITIONS FOR ANTIPSYCHOTIC RESPONSE IN DELUSIONAL DISORDER: A SYSTEMATIC AND CRITICAL REVIEW

**DOI:** 10.1093/schbul/sby016.501

**Published:** 2018-04-01

**Authors:** Alexandre González-Rodríguez, Francesc Estrada, José Antonio Monreal, Diego Palao, Javier Labad

**Affiliations:** 1 Parc Taulí University Hospital; 2 Parc Taulí University Hospital, CIBERSAM, Autonomous University of Barcelona (UAB)

## Abstract

**Background:**

Recent research in schizophrenia has revealed that there is no consensus to which is the most appropriate definition for antipsychotic response. Response rates allow the clinician to know how many subjects have responded to a specific treatment. However, once again, levels of response or the cutoff chosen have been subject of controversy, as a high variety of values have been applied in schizophrenia research. This systematic review aimed to examine all the definitions used for antipsychotic response in delusional disorder (DD), to analyze them and provide a discussion of the methodology used.

**Methods:**

A systematic computerized literature search was performed by using Pubmed, Scopus and PsycINFO databases (1990-September 2017) according to the Preferred Reporting Items for Systematic Reviews and Meta-Analyses (PRISMA) statement. In addition, reference searches were manually conducted through identified studies in order to obtain other relevant articles not previously found on the initial search strategy. The following search terms were used: [(Antipsychotic response) OR (antipsychotics) OR (treatment response)] AND [(delusional disorder)]. Studies were only included if the met our inclusion criteria: (a) be published in a peer-reviewed journal, (b) prospective or retrospective studies focusing on antipsychotic response, (c) studies assessing response in DD based on clinical judgment or clinical records, (d) studies including a definition of antipsychotic response based on assessment scales and (e) diagnostic criteria based on ICD or DSM. The literature search strategy was conducted independently by two of the authors (A.G. and F.E.). The last search was conducted on 30th October, 2017.

**Results:**

Seventy-four studies were initially identified. 39 studies were excluded after titles and abstracts were read, as they did not meet our initial inclusion criteria or met any of the exclusion criteria. 22 studies were excluded after reading the full text-document as they failed to meet our inclusion criteria or met any exclusion criteria, and 2 articles were excluded as studies for the assessment were duplicated. After the screening and selection processes, a total of 11 studies met our inclusion criteria, using different methods to define antipsychotic response in DD. Chart review (n=5) and observer-rated scales (n=6), from which 2 of them used the CGI improvement scale for assessing response, 2 studies evaluated it by mean changes from baseline to endpoint scores (PANSS, BPRS), one study combined the CGI improvement scale and mean changes from baseline scores (PANSS), and one study reported responder rates based on a scale-derived cutoff (PANSS).

**Discussion:**

A lack of consensus in the definitions of antipsychotic response in delusional disorder and a high degree of heterogeneity of the methods used are reflected on this systematic review. Although no consensus for the response definition appears to exist in delusional disorder, there is a need to better quantify the treatment response in terms of percentages of response, and linking these findings with those derived from the CGI. Recommendations from Leucht (2014) in schizophrenia would be a first step in defining response among delusional disorder patients.

